# Myocarditis and Myasthenia Gravis Induced by Camrelizumab in a Patient With Metastatic B2 Thymoma: A Case Report

**DOI:** 10.1111/1759-7714.70180

**Published:** 2025-11-04

**Authors:** Lingling Zhao, Bo Yang, Yuzhi Li, Yu Wang, Ting Zhu

**Affiliations:** ^1^ Wannan Medical College Wuhu China; ^2^ Department of Oncology The First People’s Hospital of Hefei (The Third Affiliated Hospital of Anhui Medical University) Hefei China; ^3^ Lu'an Hospital of Traditional Chinese Medicine (The Fifth Affiliated Hospital of Anhui University of Chinese Medicine) Lu'an China

**Keywords:** camrelizumab, immune checkpoint inhibitors, myocarditis; myasthenia gravis, thymoma

## Abstract

Thymoma is a rare malignant tumor originating from the thymus epithelium. In recent years, immune checkpoint inhibitors have become an indispensable treatment for cancer. However, the efficacy and adverse events of immunotherapy for thymoma have not been widely evaluated. A 53‐year‐old Chinese man who was diagnosed with metastatic B2 thymomas since March 2023. He received chemotherapy plus anlotinib for four cycles since May 5, 2023, and underwent radiotherapy from May 23, 2023 to June 30, 2023. However, the treatment was not satisfactory. Thus, we detected PD‐L1 expression in tumors; immunohistochemical examination on the tumor revealed a high PD‐L1 expression in 60% of tumor cells. He presented symptoms of palpitation, gasping, fatigue, diplopia, and eyelid ptosis. Additionally, he was found to have significantly elevated levels of serum cardiac troponin, creatine kinase, creatine kinase isoenzymes, N‐terminal pro brain natriuretic peptide, and anti‐acetylcholine receptor antibody. He was eventually diagnosed with immune‐related myocarditis and myasthenia gravis. Finally, the patient was discharged after treatment with glucocorticoids, immunoglobulin, and pyridostigmine. Although immune checkpoint inhibitors have achieved similar anti‐tumor effects in thymomas as in other solid tumors, they may be closely associated with serious immune‐related adverse events, so special caution is required when using immune checkpoint inhibitors in thymoma patients.

## Graphical abstract

Camrelizumab achieved a partial response in platinum‐refractory B2 thymoma, but triggered severe dual irAEs: myocarditis and myasthenia gravis. This case highlights the unique and serious autoimmune risks of ICIs in thymoma, mandating extreme caution in treatment decisions and patient monitoring.

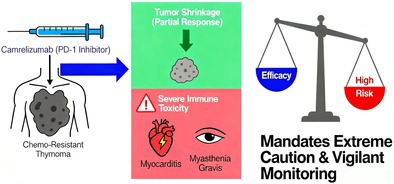

## 1 Introduction

Thymoma is a common primary tumor of the anterior mediastinum, but it is rare, accounting for about 0.2%–1.5% of all malignancies [1, 2], surgical resection is the preferred treatment, and platinum‐containing chemotherapy is the primary treatment for unresectable tumors. However, in recent years, with the growing understanding of tumor immune escape and molecular biology, along with the development of precision therapy for tumors, immune checkpoint inhibitors (ICIs) have attracted much attention. Studies have shown that PD‐1/PD‐L1 is highly expressed in 82% of thymic epithelial tumors and is more highly expressed in the more aggressive types of B2 and B3 thymomas and thymic cancers, suggesting that ICIs may have an advantage in immunotherapy for thymic epithelial tumors with higher malignancies [3]. Studies [4, 5, 6] have shown that ICIs achieve similar anti‐tumor effects in thymoma as in other solid tumors, but they are associated with a higher incidence of irAEs, particularly immune‐related myocarditis with a high mortality.

Herein, we report a case of metastatic B2 thymoma refractory to platinum chemotherapy with high PD‐L1 expression (60%), who was treated with camrelizumab, resulting in serious immune‐related adverse events (irAEs) after administration of the first cycle. To our knowledge, this is the first report of immune‐related myocarditis and myasthenia gravis caused by camrelizumab in thymomas.

## 2 Case Presentation

**FIGURE 1:**
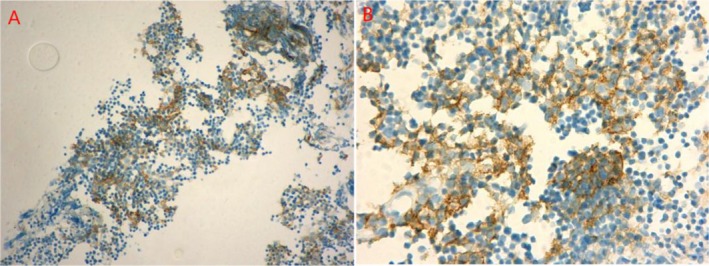
PD‐L1 expression was strong (60%) in neoplastic thymic epithelial cells. (A) Magnification X40; (B) magnification X100.

A 53‐year‐old Chinese man was diagnosed with B2 thymoma, along with metastasis to the thoracic vertebra and thyroid since March 2023. Immunohistochemical analysis shows: P63 (+), CK19 (+), Ki67 (+) 80%, CD20 (−), CD79 (+), CD3 (+), TDT (+), CD1a (+), CD117 (+). Having missed the opportunity for surgical therapy, he received chemotherapy with the EP (cis‐platinum plus etoposide) regimen plus anlotinib for four cycles since May 5, 2023, and thymoma involved the field and surrounding high‐risk lymphatic drainage areas were treated with radiotherapy from May 23, 2023 to June 30, 2023. However, the treatment was not satisfactory after four cycles of chemotherapy plus anlotinib; we suspected he was resistant to platinum chemotherapy. At the strong request of patients and their families, we detected PD‐L1 expression in tumors. Immunohistochemical examination of the tumor revealed a high PD‐L1 expression in 60% of tumor cells (Figure 1), he was treated with ICIs, camrelizumab (200 mg, q3w) on August 9, 2023.

**FIGURE 2:**
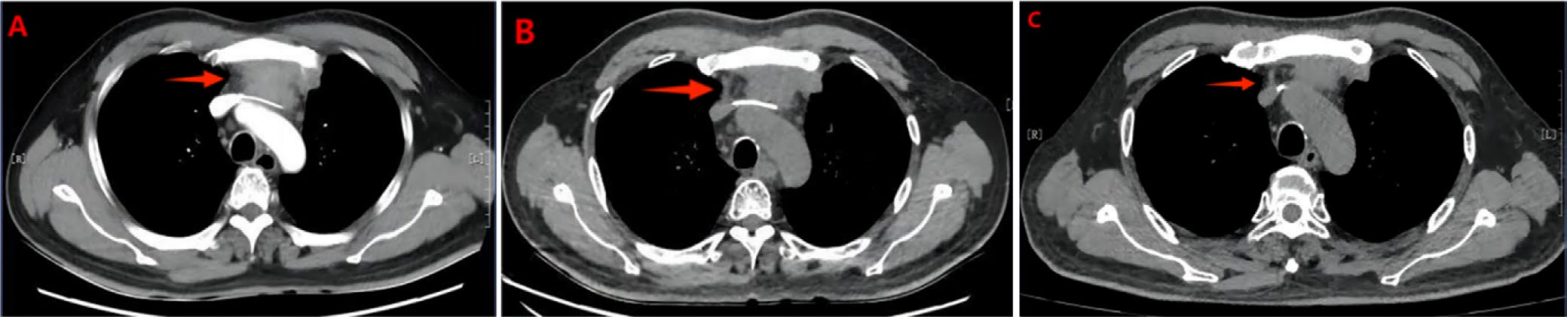
The dynamic changes of malignant lesions size as revealed by repeated chest CT scans before and after treatment. (A) Malignant lesions size before treatment. (B) Tumor lesion size after receiving 3 cycles of the EP regimen plus anlotinib. (C) Malignant lesions size after receiving camrelizumab.

**FIGURE 3:**
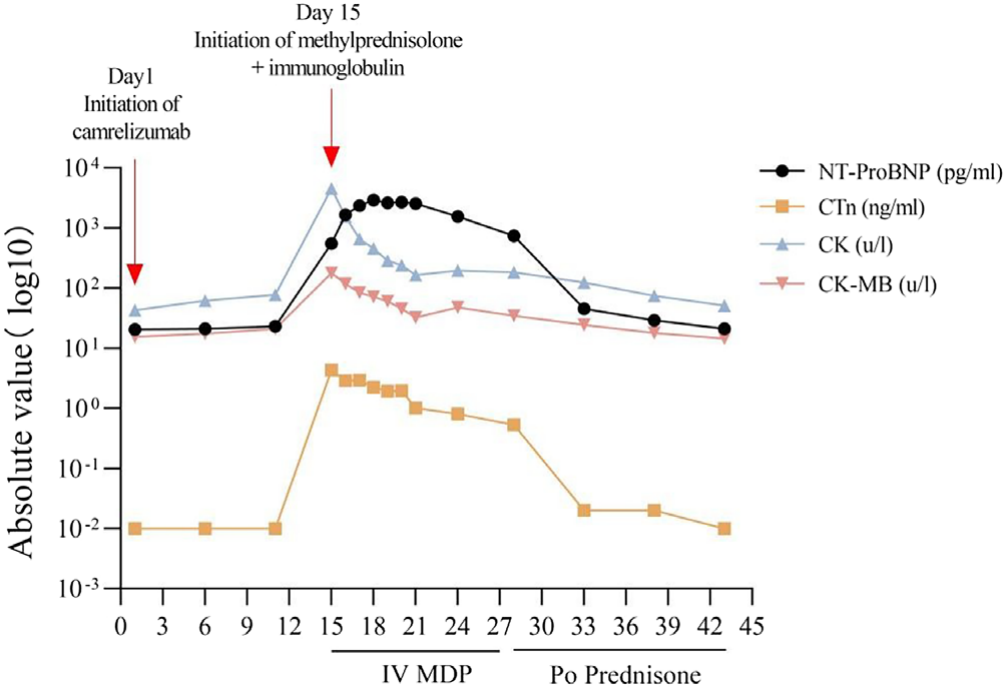
The dynamic changes in N‐terminal pro brain natriuretic peptide (NT‐proBNP) level, cardiac troponin (cTn) level, creatine kinase (CK) level, creatine kinase isoenzymes (CK‐MB) level in the patient.

After first receiving camrelizumab, the patient was re‐examined every 5 days for myocardial injury markers that were normal. On day 14, the patient appeared with symptoms of palpitation, gasping, fatigue, diplopia, and eyelid ptosis. Thereafter, the above symptoms gradually worsened, and the patient was hospitalized on day 15. Laboratory examinations showed cardiac troponin (cTn) 4.31 ng/mL (reference range 0–0.04 ng/mL), creatine kinase (CK) 4567.00 u/l (reference range 50–310 u/l), creatine kinase isoenzymes (CK‐MB) 178.00 u/l (reference range 0–25 u/l), N‐terminal pro brain natriuretic peptide (NT‐proBNP) 556.77 pg/mL (reference range 0–450 pg/mL), and anti‐acetylcholine receptor antibody 15.20 nmol/L (reference range 0–0.40 nmol/L) (Figure 3). Additionally, the electrocardiogram indicated sinus tachycardia (Heart Rate 125 bpm), and the cardiac ultrasound (left ventricular ejection fraction 71%) was normal. At the same time, we found that the tumor lesions were significantly reduced (Figure 2), and the clinical effect reached partial response (PR) according to the chest computerized tomography.

Based on the patient's clinical presentation and the above investigation, the diagnosis of immune‐related myocarditis and myasthenia gravis was suspected. He was started on the treatment with intravenous methylprednisolone (MPD, 1000 mg daily for 5 days), intravenous immunoglobulin (IVIG, 0.4 g/kg/d for 5 days), and pyridostigmine (60 mg orally four times/day) on day 15. Subsequently, the palpitation injury markers were dynamically reviewed (Figure 3), and the dose of MPD was reduced by half every 2 days. On day 33, the myocardial injury markers returned to normal levels: cTn 0.02 ng/mL, CK 124.00 u/l, CK‐MB 24.60 u/l, NT‐proBNP 45.23 pg/mL (Figure 3). Meanwhile, the symptoms of palpitation, gasping, fatigue, diplopia, and eyelid ptosis disappeared. On day 28, the patient was allowed to take orally prednisone 40 mg daily and reduced by 5 mg per week.

## 3 Discussion

Here, we report a case of metastatic B2 thymomas refractory to platinum chemotherapy who received camrelizumab, leading to a storm of irAEs.

### 3.1 Differential Diagnosis and Attribution

Symptoms required exclusion of viral myocarditis, anthracycline cardiotoxicity, paraneoplastic myasthenia gravis, and thymoma progression. Nasopharyngeal and blood viral PCR were negative, and cardiac‐MRI late‐gadolinium enhancement did not support viral myocarditis [7]. Cumulative anthracycline exposure was only 300 mg/m^2^, rarely causing acute heart failure [8]. Anti‐AChR antibodies were negative in March 2023 but became positive after camrelizumab, indicating de novo autoimmunity. Chest CT showed a partial response, ruling out thymoma progression. Consequently, camrelizumab‐associated irAE is considered the most probable cause.

Immune‐related myocarditis and myasthenia gravis are rare but life‐threatening irAEs, with acute onset and rapid progression after the initiation of ICIs. It is reported that the incidence of myocarditis and myasthenia gravis was 0.04%–1.14% and 0.12%–0.2%, respectively [9, 10]. However, emerging evidence indicates that the incidence of immune‐associated cardiovascular toxicity, particularly myocarditis, is higher than initially anticipated [11]. At present, the mechanism of irAEs caused by ICIs is still unclear [12]. Some studies have reported that it may be due to the over‐activated autoimmune cells invading normal organs of the human body and continuously attacking normal tissues and organs [13]. Thymoma can produce a large number of specific T cells against autoantigens; after T cells enter the circulating system, they can alter the T‐cell subset composition in the blood, which can cause autoimmune diseases [14]. When patients with thymoma received ICIs, the function of specific T cells against autoantigens was further activated to attack normal human tissues, which may be the mechanism of irAE in thymoma [14].

There are few data evaluating ICIs in thymomas. An open‐label phase II trial [5] enrolled 7 patients with thymoma who received pembrolizumab monotherapy. Clinical efficacy showed 2 patients (28.6%) achieved partial response (PR) and 5 (71.4%) had stable disease (SD). Meanwhile, 5 patients (71.4%) appeared grade 3 or 4 irAEs, including myocarditis in 3 patients. In a phase II trial [6], 7 patients with thymoma were treated with avelumab, including 2 who achieved confirmed PR, 2 had unconfirmed PR, 3 had SD, and 1 suffered progressive disease (PD). Five patients (71.4%) were observed to have irAEs, and 3 (42.9%) underwent grade 3 or 4 irAEs. EORTC‐ETOP NIVOTHYM phase II trial [15] recruited a total of 55 patients who were treated with nivolumab alone or with ipilimumab, including 10 patients (18%) who had B3 thymoma and 43 (78%) who had thymic carcinoma. Evaluation of efficacy showed that 7 patients (14%) achieved PR, 26 (53%) had SD, and 13 (27%) got PD. In terms of safety, 32 patients (59%) experienced at least a grade 3 irAE, and 5 cases (9%) had a grade 4 irAE. In the study by Hao et al. [16] a total of 11 patients were treated with ICIs monotherapy or combination therapy. Among them, 3 patients achieved PR, 7 had SD, and 1 had PD. The incidence of irAEs was 45.5% (5/11), of which 36.4% (4/11) were grade 3 or 4 irAEs, including myocarditis (4/11, 36.4%) and myasthenia gravis (2/11, 18.2%). Moreover, there are also many case reports of immunotherapy for thymoma. For instance, Konstantina [17], Zhong [18], Zander [19], Chen [20], Liu [21], Lu [22], et al. reported patients with thymoma who appeared severe irAEs after receiving ICIs. These data suggested that ICIs achieved similar anti‐tumor effects in thymoma as in other solid tumors, but resulted in more irAEs than other solid tumors, especially myocarditis with higher lethality. Compared with previous reports of pembrolizumab [5] and avelumab [6], this case appears to be among the earliest descriptions of camrelizumab‐associated dual irAEs (myocarditis and myasthenia gravis) in a patient with thymoma.

Immune‐related myasthenia gravis is a life‐threatening irAE that occurs early after ICI treatment, characterized by atypical symptoms, acute onset, and rapid progression. The median time from initiation of ICIs until the first myasthenia gravis symptom was 4 weeks, with a range of 6 days to 16 weeks [23]. Anti‐acetylcholine receptor antibody has been reported to be present in 80% to 85% of MG patients [24]. In patients with myasthenia gravis, ICIs should be postponed immediately. Secondly, pyridostigmine, corticosteroids, immunosuppressants, IVIG, or plasmapheresis are recommended [24, 25].

Approximately 25% of patients with Immune‐related myasthenia gravis are often complicated by myocarditis [26]. The initial symptoms of myocarditis are mostly non‐specific, such as fatigue, palpitations, and shortness of breath, which are easy to miss or misdiagnose in clinical practice. Myocarditis has a median time of onset of 27 days after starting ICI, with most cases occurring within 3 months after administration [27]. The elevation of myocardial injury markers was often earlier than the onset of clinical symptoms and positively correlated with the severity of the disease, including cTn, CK, CK‐MB, and NT‐proBNP [28]. Among them, the specificity of cTn was the highest, and the positive rate was about 90%. Elevated cTn and NT‐proBNP levels were positively correlated with the risk of death [29].

Therefore, early monitoring of patients treated with ICIs is necessary, including baseline assessment before treatment and monitoring after treatment. ICIs should be discontinued permanently in patients with grade 3 or 4 irAEs [25]. Because ICIs have a long half‐life, stopping treatment will not immediately reverse biological effects [30]. For mild‐to‐moderate cardiotoxicity, early administration of corticosteroids is recommended according to guidelines [31]. For any grade 3 or 4 cardiovascular irAEs, initiation of high‐dose intravenous corticosteroids is recommended; treatment was performed until biomarkers of cardiac injury returned to baseline levels, then dose taper over 4–6 weeks [25]. It is necessary to pay attention to the possibility of re‐exacerbation of myocarditis during the process of steroid reduction. If no improvement is found within 24 h, consider adding infliximab or anti‐thymocyte globulin [25]. In addition, mycophenolate mofetil, human immunoglobulin, and tacrolimus can also be used for immunosuppressive therapy [32].

### 3.2 Guideline Deviation Statement

AIOM 2020 and NCCN 2025 guidelines recommend against ICIs in thymoma [33, 34]. This therapy was administered as compassionate use after multidisciplinary discussion and informed consent, and the outcome reinforces the guideline warnings.

For practical guidance, if camrelizumab is nevertheless considered, we recommend baseline and pre‐cycle monitoring of cTnI, CK, ECG, and anti‐AChR antibodies, with treatment withheld should cTnI exceed 2 × ULN or a new conduction block emerge.

In our case, symptoms of myocarditis and myasthenia gravis appeared on day 10, and myocardial injury markers increased on day 15. Based on the patient's symptoms and laboratory findings, the diagnoses of immune‐related myocarditis and myasthenia gravis were considered and the clinical efficacy was PR. Finally, the patient was discharged after treatment with glucocorticoids, IVIG, and pyridostigmine. Consistent with the above studies, the patient with thymoma in our case exhibited a favorable anti‐tumor effect following the treatment with camrelizumab. Unfortunately, there were also occurrences of severe irAEs, including immune‐related myocarditis and myasthenia gravis.

## 4 Limitations

This single‐case report cannot be generalized. The rare yet severe irAEs observed reflect only this individual's response and do not constitute universal guidance for ICI use in thymoma. Additionally, long‐term follow‐up beyond 2023 is unavailable because the patient was lost to scheduled visits; thus, late‐onset toxicities or durability of response cannot be definitively assessed.

## 5 Conclusion

ICIs showed good efficacy in this single patient with metastatic B2 thymoma, but were followed by life‐threatening immune‐related myocarditis and myasthenia gravis. Current evidence does not support the routine use of ICIs in thymoma. Clinicians should carefully weigh the benefits and risks for patients, and enhance monitoring when considering the use of ICIs for thymoma treatment. We look forward to the development of large‐scale clinical trials in the future to bring new hope to the treatment of patients.

## Author Contributions

All authors contributed to proposing the treatment strategy, data analysis, drafting, or revising the manuscript. All authors reviewed and agreed on the version of the article to be published, the journal to which the manuscript has been submitted, and agreed to be accountable for the contents of the article.

## Ethics Statement

The studies involving humans were approved by the Ethics Committee of the Third Affiliated Hospital of Anhui Medical University (approval No. 202–05–186‐01, approval date: 15 September 2025). Camrelizumab was not approved by the China National Medical Products Administration (NMPA) for thymoma at the time of treatment; its use was off‐label. The studies were conducted in accordance with the local legislation and institutional requirements.

## Consent

Written informed consent has been obtained from the patient to publish this paper.

## Conflicts of Interest

The authors declare no conflicts of interest.

## Data Availability

The data that support the findings of this study are available on request from the corresponding author. The data are not publicly available due to privacy or ethical restrictions.
